# Local Oscillatory Brain Dynamics of Mind Wandering in Schizophrenia

**DOI:** 10.3390/brainsci11070910

**Published:** 2021-07-09

**Authors:** Marta Prieto, Sergio Iglesias-Parro, María Felipa Soriano, Antonio Ibáñez-Molina

**Affiliations:** 1 Psychology Department, University of Jaén, 23071 Jaén, Spain; mpamartaprieto@gmail.com (M.P.); siglesia@ujaen.es (S.I.-P.); 2 Mental Health Unit, San Agustín University Hospital, 23700 Linares, Spain; mfsoriap@gmail.com

**Keywords:** schizophrenia, Q-EEG, mind wandering, cognitive functioning

## Abstract

A number of studies have focused on brain dynamics underlying mind wandering (MW) states in healthy people. However, there is limited understanding of how the oscillatory dynamics accompanying MW states and task-focused states are characterized in clinical populations. In this study, we explored EEG local synchrony of MW associated with schizophrenia, under the premise that changes in attention that arise during MW are associated with a different pattern of brain activity. To this end, we measured the power of EEG oscillations in different frequency bands, recorded while participants watched short video clips. In the group of participants diagnosed with schizophrenia, the power in MW states was significantly lower than during task-focused states, mainly in the frontal and posterior regions. However, in the group of healthy controls, the differences in power between the task-focused and MW states occurred exclusively in the posterior region. Furthermore, the power of the frequency bands during MW and during episodes of task-focused attention correlated with cognitive variables such as processing speed and working memory. These findings on dynamic changes of local synchronization in different frequency bands and areas of the cortex can improve our understanding of mental disorders, such as schizophrenia.

## 1. Introduction

In recent years, there has been a growing interest in the study of mind wandering (MW) within the field of cognitive and clinical neuroscience. MW can be understood as a cognitive state originating from transitions between externally guided and self-generated thoughts. During MW, perceptual decoupling occurs so that sensory processing of an immediate task is reduced and attention is directed towards thoughts, images or internal memories [1]. Given the widespread presence of MW in daily life (30–50% of brain activity during wakefulness), it appears to be integrated into normal brain function [2,3]. The neural mechanisms allowing the appearance of these states, which are not related to the external task, have been a relevant object of study. Research has shown that fluctuations between internal and external attention are governed by dynamic interactions between distributed areas of the brain which operate in large-scale networks [4,5]. 

MW has been associated with the activity of the default mode network (DMN), a cortical network located along the midline of the prefrontal cortex, the rostral anterior cingulate cortex, the posterior cingulate cortex and the precuneus [6,7]. It has been suggested that this network is responsible for internally directed cognition, since it is activated during rest periods, internally oriented tasks or during thoughts, images or memories unrelated to the external task [8,9]. DMN activity, in turn, is anticorrelated with the activity of brain networks responsible for processing external stimuli [4]; that is, when the subjects are in an MW state, the DMN is more active, while activity in areas of the cortex responsible for sensory processing is reduced. In this sense, the activity of the DMN can be dissociated from the activity of other networks or specific brain areas that intervene during a cognitive or perceptual task [10]. However, some authors argue that DMN activation alone is insufficient to capture the neural base of the MW [11]. Other brain regions outside the DMN also show associations with various content and forms of MW. 

Functional connectivity studies have gained increasing interest in this area of research. It is known that the local synchrony pattern varies remarkably across the cortex [12], and it has been reported that this regional variation is associated with the hierarchical organization of information processing during human functioning [13]. A direct measure of neural synchrony is spectral power, which measures the activity of the spatial sum of the cortical responses in a local assembly [14,15,16]. Previous studies have linked episodes of MW to oscillations at particular frequencies of neuronal activity in the brain. One of these types of oscillations is the alpha rhythm [17], a neural oscillatory rhythm of approximately 10 Hz. It has been found that alpha power increases when a participant’s attention is diverted from the external task towards an internal train of thought. In other words, alpha activity is sensitive to cognitive demands, as evidenced by task-related power changes: it is suppressed in response to sensory stimulation [18] and increased during periods without stimulation [19,20]. Numerous studies have shown this role of alpha power in the inhibition of sensory processes to protect thinking from ongoing distraction [21,22,23,24], and it is considered to be a promising measure to characterize MW [25]. These results are consistent with numerous previous findings that associate increased alpha power with MW in frontal, occipital and posterior areas of the brain [20,26,27,28,29,30], although some studies have found the opposite pattern [31,32]. 

MW states are not exclusively related to alpha power. When considering the oscillatory activity of an EEG, the dynamics of other frequency bands could also be of particular interest. For example, during MW, an increase in theta and a reduction in beta power have been observed compared to episodes of focused attention [33]. In this line, Kirschner et al. [10] also found a pattern of differential oscillatory power changes during periods in which attention was directed internally and periods in which attention was focused on an external task in each of the frequency bands: theta, alpha and gamma. Taking these data as a whole, the study of the dynamic changes of power in the different frequency bands and in different areas of the cortex can be a valuable tool for the study of fluctuations between MW and external attention. 

From a clinical point of view, the dysfunctional properties associated with MW are closely related to neuropsychiatric disorders [34,35]. Recent research [36,37] has reported that patients with schizophrenia exhibited states of MW more frequently than healthy controls. This greater frequency of MW has been explained by a frequent disconnection of attention from external events accompanied by an excessive focus on the inner world [37,38,39,40]. Dysfunctional properties associated with MW in schizophrenia may be mediated by deficits at the neural level. For example, there is evidence describing aberrant dysfunctional connectivity patterns and hyperactivity both in the DMN, as well as between this network and other neural networks [41]. The dysfunctional brain dynamics in schizophrenia could be responsible for diverting attention resources when patients try to perceive, think or act in the external world [8,42], leading to an increase in MW episodes.

Hence, schizophrenia can be characterized by alterations in brain connectivity during states of rest and during cognitive tasks [43,44,45]. Some recent research has used local synchrony to explore local brain activity in schizophrenia [41,46,47,48]. These studies are based on an extensive bibliography that reports alterations related to neural synchrony of variable frequency, anatomical regionalization and cognitive relevance [49,50]. Xu et al. [48] found that, compared to healthy controls, brain activity in patients with schizophrenia is characterized by decreased local synchrony in the somatosensory, posterior parietal and occipital cortices, but increased synchrony mainly in the medial temporal and medial prefrontal cortices. Local alterations in synchrony suggest a disrupted balance in the local functionality of the entire brain network and are characterized by alterations in the power, or amplitude, of local oscillations within a brain region [51] that may be present in the first psychotic episode [52]. The severity of these deficits correlates with the severity of the symptoms [53], which suggests that abnormal brain dynamics at the neural level may be related to the cognitive and behavioral deficits associated with schizophrenia. For example, patients with schizophrenia show reduced gamma and theta frequency oscillatory power in the frontal areas, and this reduction is related to the performance of the cognitive tasks of working memory [54], whereas in healthy controls, these types of tasks induce increases in oscillatory power [55]. Adams et al. [43] demonstrated that during memory retrieval for a spatial memory task, increased theta power in the left-medial temporal lobe is altered in schizophrenia. Along these lines, altered oscillatory activity has been observed at high frequencies in schizophrenia patients [51]. For example, Grützner et al. [56] described deficits in gamma power in occipital areas during a visual processing task. However, these disturbances cannot be measured solely during a specific task. In states of rest, in the absence of a specific external stimulus or task, deficits in gamma frequency oscillatory power have been described in cortical areas corresponding to the DMN [51,57] and increases in theta and alpha frequency bands in the posterior cingulate cortex in schizophrenia patients [44].

These findings, combined with previous results [36], confirm differences in EEG modulation between patients with schizophrenia and healthy controls and lead us to the subject of the present work. Iglesias-Parro et al. [36] found a group modulation by electrode sites in which the EEG complexity pattern in the frontal and temporal areas was lower for patients than controls. Furthermore, an interesting finding was that in the group of patients, no differences were found between episodes of MW and episodes of focused attention regarding EEG complexity, which suggests that, at a physiological level, the thinking of patients does not differ between internally directed care and externally oriented attention. Our research allowed us to extend these results by exploring the neural correlates in local EEG synchrony that accompany MW and attention to an external task in schizophrenia. Specifically, we intended to characterize the differences between both states according to the power in each frequency band and cortical location. The approach we presented measured the oscillatory power in different frequency bands evoked by a specific task, in which MW episodes were recorded while participants (schizophrenia patients and healthy controls) watched short video clips. Based on previous data, we expected to find differences in the oscillatory dynamics that accompany the fluctuations between MW and external attention in different cortical regions.

## 2. Materials and Methods

### 2.1. The Experimental Task

Participants watched 4 video clips extracted from non-blockbuster Spanish films. Each clip was 5 min long: in two of them, visual and auditory information matched (they came from the same movie), while in the other two, visual and auditory information did not match (they came from different movies). To find out whether the participants were familiar with the movies, we asked them at the end of the task. Only one participant reported vaguely remembering one of the movies. See [36] for a more detailed description of the procedure for obtaining the clips and the experimental control.

### 2.2. Cognitive Evaluation

To measure the putative cognitive impairment of the participants in the SZC group, the Spanish version of the Screen for Cognitive Impairment in Psychiatry (SCIP-S) was employed [58]. The SCIP-S is a screening instrument to assess cognition in clinical samples, showing good reliability and adequate concurrent validity with other well-known neuropsychological tests [59]. It consists of five subscales, and in about 15 min provides a score of working memory, immediate and delayed verbal learning, verbal fluency and psychomotor speed, as well as a global score. Furthermore, we employed the Spanish version [60] of the D2 Test of Attention [61], in which individuals have to search for target items among the distractors. From D2, it was possible to calculate the total number of responses (TR), total number of hits (TH), omitted elements (O), commissions (the number of irrelevant elements marked) (C), total test effectiveness (TOT) (TR−(O + C)), concentration index (CON) (TH−C), the item with a greater number of tried elements (TR+), the item with a lower number of tried elements (TR−) and the variation index (V) (the difference between TR+ and TR−).

### 2.3. Symptom Scale

The Spanish version [62] of the Positive and Negative Syndrome Scale (PANSS, [63]) was employed to measure psychopathology. It can be divided into two subscales of 7 items: the positive subscale (M = 14, SD = 6.08) and the negative subscale (M = 18.85, SD = 7.60). PANSS also provided a general measure of psychopathology (M = 32.41, SD = 9.33).

### 2.4. Procedure

Data collection was carried out in two sessions in a soundproofed room at San Agustín University Hospital. In the first session, after signing an informed consent form, the participants sat about 70 cm from the center of a computer screen. The experimenter then placed a 31-active-electrode assembly on the 10–20 system. All electrodes were re-referenced to both mastoids. The impedance of all electrodes was kept below 5 kOhm. Signals were recorded at a frequency of 500 Hz. Subsequently, instructions were given to the participants about the experimental task. They were told they would be shown several movie segments and that after each they would be asked whether they paid attention to the video content or to unrelated content in their minds (MW). We emphasized that there were no right answers; therefore, it was not necessary for them to make an extra effort to pay attention. Approximately every 60 s, the video was paused, and participants were asked via a screen message to verbally report whether they were attending to the video content (on task) or were distracted (MW state). The investigator, who remained outside the participant’s visual field, recorded the verbal responses by entering a numerical code into the EEG recording. Participants had 10 s to respond, then the message disappeared, and the video continued until the next pause. In the second session, participants underwent cognitive assessment (SCIP-S and D2). Afterwards (in case the participant was a patient), a clinician administered the PANSS.

### 2.5. Participants

The sample used in this work was part of a large investigation designed to compare internally guided cognition in schizophrenia patients and healthy controls. Although the results to be presented in this paper have not been previously published and the research questions are original, the sample description and the cognitive functioning assessment procedures used have been described in detail elsewhere [36].

The Hospital Human Research Ethics Committee approved the study. Part of the sample for this study consisted of 22 adults from the Mental Health Unit of the San Agustín University Hospital in Spain (hereinafter, SCZ Group). The criterion for participation was an ICD-10 diagnosis of schizophrenia, i.e., psychotic or schizoaffective disorder. The ages ranged from 23 to 53 years (M = 36.54; SD = 10.23). Seven of the participants were female, and all were right-handed. Of the participants, 5 had primary education, 13 had secondary education and 7 had higher education. The average time in treatment since diagnosis was 14.68 years (SD = 9.53, min = 1, max = 35). All patients were receiving antipsychotic treatment: 21 with typical and 1 with atypical antipsychotics. Due to differences in dosage, they were transformed into chlorpromazine equivalents (M = 676.14 mg, SD = 359.43 mg) so that comparisons on a common scale could be made.

The control group was composed of 23 adults recruited from the University of Jaén, the staff of the San Agustín University Hospital and among volunteers at an adult school in Jaén (hereinafter the CTR Group). The ages ranged between 23 and 57 years (M = 38.87; SD = 11.86). Of the participants, 4 were women and 21 were right-handed, and 2 had primary education, 14 had secondary education and 7 had higher education. There was no significant association between sex and group (*χ*^2^ (1, N = 45) = 0.91, *p* = 0.33) or between group and education level (χ^2^ (2, N = 45) = 2.12, *p* = 0.34). The groups did not differ significantly with respect to age (*t* = 0.70, *p* = 0.48).

### 2.6. EEG Data Analyses

**Figure 1:**
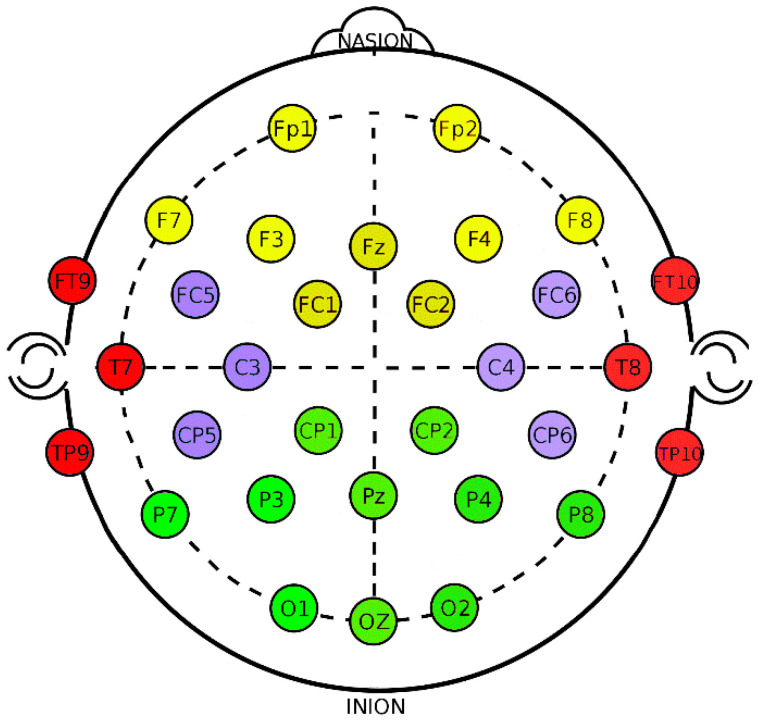
Electrodes and regions of interest used in the study. The grouping of electrodes are indicated with colors: Central (C; purple), Frontal (F; yellow), Posterior (P; light green) and Temporal Left (T; red).

Data processing was carried out with EEGLAB [64] and MATLAB [65]. A bandpass filter between 1 and 30 Hz was applied. For each participant, we took 20 clean EEG segments, with a duration of about 50 s, corresponding to each of the 20 video sequences they had watched. Each segment was labeled according to the participants’ MW or on-task response. Blinks, eye movement, muscle activity and other EEG data were removed using infomax ICA [66]. After removing noisy segments, the resulting EEGs were used to obtain the power in each frequency band. We applied the fast Fourier transform (FFT) to obtain the spectrum of the EEG signals. The electrodes were grouped into areas of interest (see Figure 1).

## 3. Results

We conducted a mixed-effects ANOVA on EEG’s power because we had different numbers of MW and on-task responses across experimental conditions. Analyses were performed in R (R Core team, 2014) using the *lmer* function of the *lme4* package [67]. Predictor variables included the within-participants answer (MW vs. on-task), area (frontal, central, temporal and posterior) and frequency (delta, theta, alpha, beta and gamma), and the between-participants factor group (SCZ vs. CTR) and all the interactions. Random intercepts were included. Residuals showed departures from normal (Lilliefors test: *D* = 0.09; *p* < 0.01), but due to the robustness of the linear model, we proceeded with the mixed model [68,69]. Levene’s test for homogeneity of variance was significant (*F*(79, 3015) = 8.63; *p*  <  0.01) and visual inspection of the residuals confirmed more dispersion of the power in the lower frequency bands. Due to our unbalanced data, we used the type III sum of squares, and because of our relatively small sample, we used the restricted maximum likelihood as the estimation procedure [70]. We also used the Satterthwaite approximation to estimate denominator degrees of freedom for the *F* statistics correction to account for heterogeneous variances [71].

**Figure 2:**
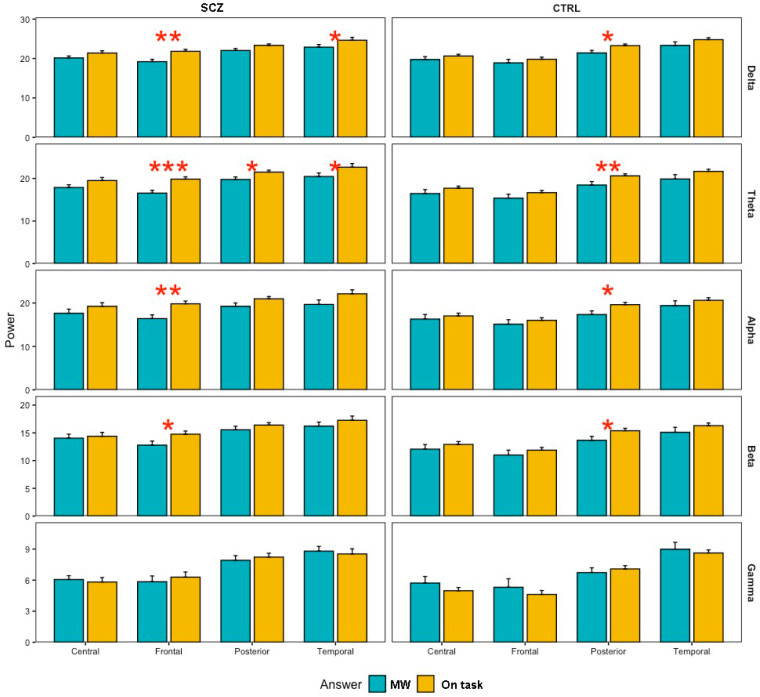
Power across answer (on-task vs. MW) for different frequency bands (delta, theta, alpha, beta and gamma) depending on group (Schizophrenia—SCZ vs. Control—CTR) and region (Central, Frontal, Posterior and Temporal). Red asterisks represent significance: *** *p* < 0.001, ** *p* < 0.01, * *p* < 0.05.

The results (see Figure 2) showed no significant main effect for group (*F* = 2.85; *p* =  0.09) but a significant main effect for answer (*F* = 33.56; *p*  <  0.01), area (*F* = 121.25; *p* < 0.01) and frequency (*F* = 418.35; *p* < 0.01). Although the interaction group by area was not significant (*F* < 1), the interaction group by answer (*F* = 5.24; *p* < 0.05), answer by area (*F* = 4.68; *p* < 0.01) and group by frequency (*F* = 7.75; *p* < 0.01) were significant. Group by answer by area (*F* = 4.45; *p* < 0.01) and group by answer by frequency (*F* = 5.24; *p* < 0.05) were also significant interactions. None of the others were significant (all *F* < 1).

To explore simple effects, we conducted non-parametric pairwise comparisons using a Wilcoxon test between the frequency power of on-task vs. MW across experimental conditions. A summary of significant tests is shown in Figure 2. More detailed information can be found in Table 1. As can be seen, power in the on-task trials for schizophrenia patients was significantly higher than for the MW trials, mainly in the frontal region (and temporal for the slowest frequencies: delta and theta). However, in the group of healthy controls, the differences between on-task vs. MW power occurred exclusively in the posterior region.

**Figure 3:**
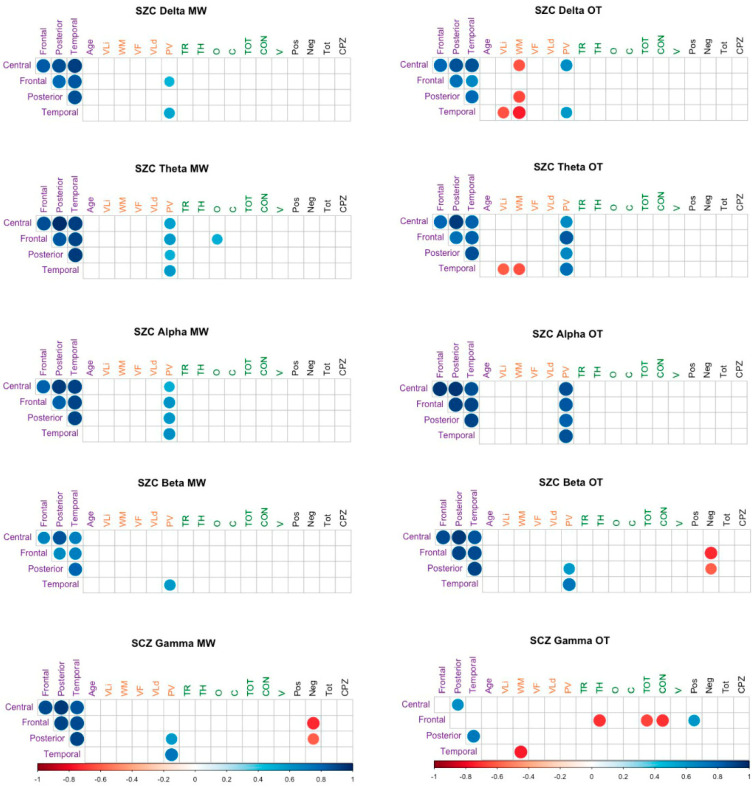
Spearman correlation coefficients. TR (D2): Number of responses, TH (D2): Hits, C (D2): Commissions, O (D2): Omissions, TOT (D2): Total score, CON (D2): Concentration, V (D2): Variation Index, Vli (SCIP): Immediate Verbal Learning, WM (SCIP): Working Memory, PV (SCIP): Processing Speed, VLd (SCIP): Delayed Verbal Learning, VF (SCIP): Verbal Fluency, Pos: Positive symptoms of PANSS, Neg: Negative symptoms of PANSS, Tot: General Psychopathology of PANSS, CPZ: Chlorpromazine equivalent.

To explore a possible relationship among power in each scalp region, measures of attention (D2), cognitive functioning (SCIP), positive and negative symptoms (PANSS) and the chlorpromazine (CPZ) dose in the patient group, we calculated the Spearman correlation coefficient among these variables. The false discovery rate (FDR) procedure [72] was used to control the increase in type I errors due to the number-of-significance test. The results are shown in Figure 3, and only significant coefficients are shown. We found significant and positive correlations between theta and alpha amplitude and processing speed both in MW and on-task participants. We also found a significant and negative correlation between the amplitude of slower rhythms and working memory, especially in the temporal region when participants were on-task.

**Figure 4:**
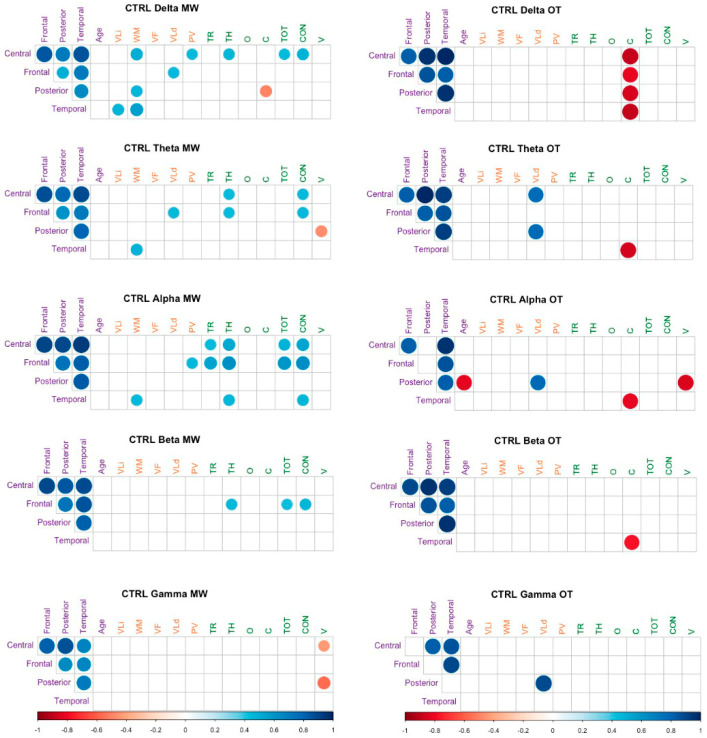
Spearman correlation coefficients. TR (D2): Number of responses, TH (D2): Hits, C (D2): Commissions, O (D2): Omissions, TOT (D2): Total score, CON (D2): Concentration, V (D2): Variation Index, Vli (SCIP): Immediate Verbal Learning, WM (SCIP): Working Memory, PV (SCIP): Processing Speed, VLd (SCIP): Delayed Verbal Learning, VF (SCIP): Verbal Fluency.

Similarly, in the control group, we calculated the correlation between power in each scalp region and our measures of attention (D2) and cognitive function (SCIP). The FDR procedure [72] was used to control the increase in type I errors due to the number of significance tests. The results are shown in Figure 4. It is noteworthy that when participants were on-task, a significant negative correlation was found between commission errors in almost all rhythms, especially in the temporal region. Likewise, in alpha, there seems to be a relative disconnect between the power of the posterior region and that of the central and frontal regions. On the other hand, when participants were in MW, power in frontal and central regions seemed to be associated with measures of attention.

## 4. Discussion

The present study was aimed at characterizing the neural substrates of MW associated with schizophrenia under the premise that the changes in attention that arise during MW are associated with a different pattern of brain activity. Specifically, we studied the changes in the spectral power of the different frequency bands (delta, theta, alpha, beta and gamma) in different scalp regions (frontal, central, posterior and temporal) during periods of MW and task-focused attention in the context of an undemanding task.

Using an online experience sampling probe paradigm during the experimental task, in conjunction with brain electroencephalography (EEG) signal recording, we observed that MW episodes were associated with reduced overall spectral power compared to episodes of task-focused attention. These data suggest a local desynchronization during MW episodes in the context of the ongoing external task. This idea is supported by the authors of [31], who argued that MW is correlated with decreased power in response to external task stimuli. According to these authors, MW modulates the oscillatory mode of the brain during the external task since it corresponds to a situation in which the processing of external stimuli or inputs is less stable, such that more cortical processing is required to remain attentive to the task [31].

Specifically, our results suggested that, in contrast to task-focused attention, the MW neuronal signature was characterized by weaker local functional connectivity in most frequency bands for both groups. Furthermore, these differences between states were especially present in schizophrenia patients for the slower oscillations (delta and theta frequency bands) at different scalp sites. These data contrast with the results of a recent study with healthy people [73], which suggests that MW neural activity, compared to task-focused attention, is characterized by a general increase in alpha, theta and delta frequencies. In this sense, several recent investigations were focused on examining the dynamics of different frequency bands during episodes of MW and task-focused attention in healthy populations. However, the literature on electrophysiological correlates of MW is rather inconsistent. While some studies found increases in the power of some frequency bands to be associated with episodes of MW, others found the opposite pattern. For example, several studies with healthy controls considered alpha power to be an index of non-task-related thinking (MW) when the task involved responding to external stimuli [20,26,30,73]. However, our results did not support this interpretation, but rather the opposite: a decrease in alpha power during mental distractions or attention lapses. This inconsistency may be explained by a number of variables, such as the choice of signal processing methods, cognitive demands of the external task or the assessment strategy used to explore MW episodes. For example, our results contrasted with those from the study by Compton et al. [20], who used a Stroop Task using a thought-probe paradigm to capture MW episodes. They found enhanced alpha power during MW compared to task trials. Instead, our results were consistent with previous studies [31] that used the sample-probe method to investigate MW during an undemanding surveillance task. Baird et al. [31] found a decrease in alpha and beta spectral power during MW episodes, compared to states of task-focused attention. Thus, it is possible that variables such as the method used to evaluate MW, or the processing demands of the main task, could explain inconsistent results. 

The second relevant finding in our study was that, in the group of patients, task-focused attention was significantly greater than during MW, mainly in the frontal region (and temporal region for delta and theta frequencies). However, in the group of healthy controls, the differences between episodes of task-focused attention and MW occurred exclusively in the posterior region. These findings supported the role of specific cortical regions in fluctuation between internally generated thoughts and external task-related thoughts in patients and controls. These cortical activation changes could be responsible for the greater susceptibility to MW associated with schizophrenia [36,37]. In patients, the differences between the two states were mainly located in the frontal and temporal regions, which are closely related to cognitive and clinical symptoms, especially with the deficient mechanisms of attentional control responsible for inhibiting interference (external or internal) with the task. These findings corroborated current theoretical frameworks that emphasize the role of the temporal and frontal areas in supporting self-generated thoughts (MW) in clinical populations [48,74,75]. Furthermore, according to Thézé et al. [76], abnormal brain activation in these regions in patients may suggest greater cortical processing to connect to the task. However, the results for the controls showed that differences between MW and task-focused attention were associated with the connectivity of posterior areas of the cortex. Our findings are consistent with other studies [27] that highlight the role of the alpha frequency in posterior brain regions for different forms of internally directed attention. It should be considered that the oscillatory activity of the EEG is not necessarily a uniform phenomenon; that is, it can be associated with different cognitive processes, which can explain the variation between different topographic regions of the cortex as well as the differences between groups.

Finally, our results revealed significant and positive correlations between theta and alpha amplitude and the processing speed in the group of schizophrenia patients, both in MW and in episodes of attention to the task. This relationship is consistent with previous research [77,78] that linked processing speed with increases in the oscillatory power of alpha and theta frequencies. Furthermore, our results are consistent with those of a recent study with people with ADHD [79]. These authors found that theta power and alpha power were associated with the ability to inhibit spontaneous and task-irrelevant thoughts as well as with the greatest variability in reaction times. On the other hand, there was a significant negative correlation between the amplitude of the slower rhythms and working memory, especially in the temporal region when the patients concentrated on the external task. These data are in agreement with numerous EEG studies that found a reduction in theta power related to tasks involving working memory in those with schizophrenia [80,81]. In the group of healthy controls, the correlations reflected a relative disconnect between the power of the posterior region and that of the central and frontal regions in the alpha frequency band when they were paying attention to the task. These results support previous evidence describing functional cortical inhibition in ongoing processing that is reflected in the oscillatory activity of the alpha band [23,82]. Specifically, this framework predicted that optimal task performance correlates with a decrease in alpha activity in regions relevant to task processing and an increase in regions irrelevant to the task.

## 5. Conclusions

This study provided new insights into neural markers of MW and task-focused states in patients with schizophrenia. We found that MW was associated with a decrease in the oscillatory power of the delta, theta, alpha and beta bands, and these findings help to clarify the differences in MW between people with schizophrenia and healthy people, as well as which cortical regions intervene during dynamic changes between the two states. Future research should continue the study of oscillatory activity during MW in schizophrenia and related mental disorders.

## Figures and Tables

**Table 1:** Probability values for pairwise comparisons between on-task and MW.

Region	Frequency	Group	*p*-Value	Region	Frequency	Group	*p*-Value
Central	Delta	CTR	0.19	Posterior	Delta	CTR	0.01
		SCZ	0.06			SCZ	0.11
	Theta	CTR	0.18		Theta	CTR	0.01
		SCZ	0.07			SCZ	0.03
	Alpha	CTR	0.48		Alpha	CTR	0.16
		SCZ	0.18			SCZ	0.08
	Beta	CTR	0.39		Beta	CTR	0.03
		SCZ	0.38			SCZ	0.31
	Gamma	CTR	0.47		Gamma	CTR	0.61
		SCZ	0.98			SCZ	0.90
Frontal	Delta	CTR	0.28	Temporal	Delta	CTR	0.07
		SCZ	0.00			SCZ	0.03
	Theta	CTR	0.16		Theta	CTR	0.08
		SCZ	0.00			SCZ	0.02
	Alpha	CTR	0.46		Alpha	CTR	0.28
		SCZ	0.00			SCZ	0.06
	Beta	CTR	0.46		Beta	CTR	0.20
		SCZ	0.03			SCZ	0.11
	Gamma	CTR	0.60		Gamma	CTR	0.88
		SCZ	0.60			SCZ	0.82

## Data Availability

Data are available upon request from the corresponding author.
